# TLC densitometric approach for concurrent determination of quinary mixture for treatment of migraine with appraisal to method greenness and whiteness

**DOI:** 10.1038/s41598-024-79518-5

**Published:** 2024-12-16

**Authors:** Marco M. Z. Sharkawi, Marwa A. Ahmed, Nehal F. Farid, Nada S. Abdelwahab

**Affiliations:** 1 Pharmaceutical Analytical Chemistry Department, Faculty of Pharmacy, Beni-Suef University, Al Shaheed Shehata Ahmed Hegazy Street, Beni-Suef, 62514 Egypt; 2 Forensic Chemist, Forensic Medicine Authority, Bayram El Tounsy St., El sayeda zeinab, CAIRO, Egypt

**Keywords:** TLC densitometry, Ergotamine, Phenobarbital, Caffeine, Dipyrone, Meprobamate, Chemistry, Analytical chemistry

## Abstract

A new accurate, precise thin layer chromatographic (TLC densitometric) approach was developed for the simultaneous analysis of ergotamine tartrate (ERG), phenobarbital sodium (PHEN), caffeine anhydrous (CAF), dipyrone sodium (DIP) and meprobamate (MEP) in their pure form and in their combined formulation; (MIGRANIL tablets). TLC separation depended on using a stationary phase of TLC plates F_254_ (20 × 10 cm) and using a developing system of ethyl acetate: methanol: n- hexane (8:2:3, by volume) with UV scanning at 254 nm for ERG, PHEN, CAF and DIP. For MEP, detection was done at 560 nm after spraying with 0.3% ethanolic solution of para -dimethylaminobenzaldehyde (PDAB) and heating at 110 ºC for 30 min. Analysis was performed in the concentration intervals of 0.06-2, 0.15–5 µg/band for ERG and PHEN, respectively; while for CAF, DIP and MEP it was done in the ranges of 0.3-9, 0.5–10, 1–100 µg/band, respectively. Moreover, the method was used for the quantitation of the five studied drugs in their unique formulation and the recovery% was calculated and found to be 102.04, 100.73, 100.65, 99.91, 1nd 101.77% for ERG, PHEN, CAF, DIP, and MEP, in order and all these outcomes proved excellent selectivity of the method. Other validation parameters were tested following ICH guidelines. Accuracy ranged from 98.02 to 100.53% while the obtained %RSD of interday day precision ranged from 1.215 to 1.940%. The environmental impact and performance of the method was tested with several tools; Analytical eco-scale, green analytical procedure index (GAPI), Analytical GREEnness Metric Approach (AGREE), Blue applicability grade index (BAGI), and RGB additive color model. Results ensured minimum hazardous effect on health and environment. This is the first analytical approach developed for simultaneous analysis of the quinary mixture which can be easily applied in any analytical laboratory.

**Supplementary Information:**

The online version contains supplementary material available at 10.1038/s41598-024-79518-5.

## Introduction

Strong headache is a common side effect of the neurological condition; migraine. The headache occurs in spurts and occasionally is accompanied by light sensitivity, vomiting and nausea^1^. Numerous comorbidities, such as anxiety, irritable bowel syndrome, overactive bladder, sleep difficulties, and obsessive-compulsive disorders are also linked to migraine^2^. Five compounds; ergotamine tartrate (ERG), phenobarbital sodium (PHEN), caffeine anhydrous (CAF), dipyrone sodium (DIP) and meprobamate (MEP); are formulated in single tablets dosage form which can control and decrease the migraine attack.

Ergotamine tartrate (ERG) is stated in the British Pharmacopeia as an ergot alkaloid^3^. It is utilized in the prevention or treatment of vascular headaches, including cluster headaches and migraines. While PHEN is a barbituric acid derivative and nonselective central nervous system depressant which helps in treating the stress associated with migraine and is prescribed to treat anxiety, seizures, and sleeplessness disorders^4^. CAF is a methylxanthine alkaloid^4^ that can boost the analgesic efficacy of the main ingredient in analgesic formulations by up to 40% because of its adjuvant properties^5^. DIP, also called analgin, is regarded as a prodrug and is frequently used as an analgesic and antipyretic^6^. MEP binds to GABA A receptors so produces sedation and alters perception of pain and has a skeletal muscle relaxant and tranquilizer effect^7^.

There were several previous publications for the determination of each analyte of this co-formulated mixture either as a single drug or with other components. There were publications that analyzed some of them in one mixture. ERG, CAF and DIP were determined together by HPTLC^8^, spectrophotometry^9^, and spectrofluorimetric method combined with chemometry^10^. Only one method was published for the analysis of DIP, CAF and PHEN together with paracetamol and codeine by HPLC^11^. Different combinations containing ERG and CAF in presence of other drugs were analyzed by various methods including TLC^12,13^, HPLC^14–17^, and capillary electrophoresis^18^.

Moreover, some approaches were published for quantification of DIP and CAF in the presence of other drugs as HPLC^19–21^ and electrophoresis^22,23^. Additionally, DIP together with PHEN and Khellin were analyzed by HPLC method^24^, whereas, PHEN and CAF along with phenacetin and aspirin were determined by spectrophotometry^25^. While HPLC methods were published for their analysis either with acetylsalicylic acid and paracetamol^26^ or with other drugs in presence of excipients^27^. In contrast, PHEN and MEP were determined by UPLC- MS^28^. Finally, CAF and MEP with morphine, 6-monoacetylmorphine and cyamemazine were determined by GC- MS^29^.

From the mentioned literature review, no publications were found for the concurrent determination of the quinary mixture under study. The challenge in this combination is the presence of MEP which lacks a chromophoric group making it difficult to be directly determined using traditional methods of analysis.

Most endeavors to diminish the environmental ramifications of chemical processes concentrate on using more ecologically friendly solvents or on getting rid of solvents using fewer reagents and auxiliaries. To develop a totally green analytical method is almost impossible due to the scarcity of available green solvents. Therefore, it is necessary to evaluate the environmental impact and applicability of any developed method using different metrics like Analytical Eco-scale^30^, green analytical procedure index (GAPI)^31^, Analytical GREEnness Metric Approach (AGREE)^32^, Blue applicability grade index (BAGI)^33^, and RGB additive color model^34^.

Hence, this work aims to establish for the first time a new, sensitive, time saving and an economical method for the concurrent analysis of the five studied drugs; ERG, PHEN, CAF, DIP and MEP. The developed method has the advantages of saving energy and consuming less amount of solvents, hence it has a low environmental impact. Additionally, the work is optimized and validated according to instructions of the International Conference on Harmonization (ICH) Guidelines^35^.

## Experimental

### Instruments

- A TLC linomat V sample applicator was used along with A 100-µl syringe (CAMAG; Muttenz, Switzerland) for the application of samples on TLC plates (20 cm x 10 cm) coated with 0.25 mm thick silica gel 60 F_254_ (MERCK, Germany).
- Chromatographic development was performed in a tank using an eluent consisting of ethyl acetate: methanol: n- hexane (8:2:3, by volume).
- Plates were scanned using TLC Scanner 3 densitometer (CAMAG), with wavelength settings at 254 nm for ERG, PHEN, CAF, and DIP, and 560 nm for MEP. The winCATS program (V 3.15, CAMAG) was used to run the scanner, which had a slit size of 3.00 × 0.45 mm and a scanning speed of 20 mm/s.
- A UV lamp with a short wavelength of 254 nm (VL-6.LC; Marne-la-Vallee Cedex 1, France) was used during method optimization.

### Chemicals and samples

The used chemicals and solvents were of analytical grade.

- Ethyl acetate, ethanol, methanol, n-hexane, hydrochloric acid (HCl) were obtained from El NASR Pharmaceutical Chemicals Co, Cairo, Egypt.
- p-dimethylaminobenzaldehyde (PDAB) was supplied from Sigma-Aldrich, Chemie, Germany.
- Pure raw material samples of ERG (100.20%), PHEN (100.53%), CAF (100.06%), DIP (100.07%), MEP (98.02%) (purity was evaluated according to the developed method), were kindly obtained from The NILE Co. for Pharmaceuticals and Chemical Industries, Cairo, Egypt. Their purity was additionally evaluated according to methods of assay reported in USP^36^ and was found to be 99.55, 98.90, 100.41, and 99.32% for ERG, PHEN, CAF, and MEP, in order.
- MIGRANIL tablets (batch no. t095003) were produced by The NILE Co. for Pharmaceuticals and Chemical Industries, Cairo, Egypt. Each tablet contains 1 mg ergotamine tartarate, 10 mg phenobarbital sodium, 50 mg caffeine, 150 mg meprobamate, 200 mg dipyrone.

### Prepared solutions

#### Standards solutions

Stock solutions: 0.01 g each of ERG, PHEN, CAF and DIP, 0.1 g of MEP were precisely weighed and transferred into five separate 10 mL measuring flasks. Methanol was added, mixed well and then completed to the volume to reach 1000 µg/mL each of ERG, PHEN, CAF and DIP and 10,000 µg/mL of MEP.

Working solutions: 1 mL each of ERG, PHEN, CAF, DIP, MEP were accurately taken from their corresponding stock solutions into five separate 10 mL measuring flasks, then the volume was completed with methanol to prepare diluted solutions of 100 µg/mL each of ERG, PHEN CAF, and DIP and of 1000 µg/ mL for MEP.

#### Sample solution

After ten MIGRANIL tablets were weighed, finely ground, and thoroughly mixed, a precisely weighed quantity of the powder equivalent to 0.5 mg ERG, 5 mg PHEN, 25 mg CAF, 75 mg MEP, and 100 mg DIP was transferred into a 50 mL calibrated flask, 30 mL methanol was added, thoroughly mixed, and the flask was filled to the mark with methanol. The resulting solution was sonicated for 15 min. and then filtered. The obtained stock sample concentration was 0.01 mg/mL ERG, 0.1 mg/mL PHEN, 0.5 mg/mL CAF, 1.5 mg/mL MEP and 2 mg/mL DIP. Diluted sample was prepared from the stock sample solution by transferring 2 mL to a 10 mL volumetric flask using methanol.

#### PDAB solution

0.3% ethanolic solution of PDAB was prepared by dissolving 0.3 g of PDAB in 90 mL ethanol and 10 mL concentrated HCl. This reagent was used as a spray for visualization of MEP on TLC plates^37,38^.

### Procedure

#### Construction of calibration graphs

Sample solutions in the concentration ranges of 6–200 µg/mL ERG, 15–500 µg/mL PHEN, 30–900 µg/mL CAF, 100–10,000 µg/mL MEP and 50–1000 µg/mL DIP were prepared in methanol in five separate series of 10 mL measuring flasks using their formerly prepared stock and working solutions. 10 µL of each sample was applied in triplicate as bands of 3 mm width to TLC plates (20 × 10 cm). Development was done using an eluent composed of ethyl acetate–methanol–n-hexane (8:2:3, by volume) at 25 °C. Plates were removed from the jars and were air dried after which scanning was performed at 254 nm. After that plates were sprayed with 0.3% ethanolic solution of PDAB and heated for 30 min at 110 º C then scanning was repeated at 560 nm. Peak areas were recorded and were plotted versus the corresponding concentrations for each component to establish the calibration curve and calculate the regression parameters.

#### Application to dosage form

10 µL of each of the stock and diluted sample solutions were applied each in sextuplets, to TLC plates and the process under construction of calibration curve was followed. Peak areas were recorded and concentrations were computed from the corresponding regression equations. Moreover, standard addition was performed on three different levels; 80, 100 and 120% to test the accuracy of the method.

## Results and discussion

After extensive reviewing in the literature, no publications were found for the concurrent analysis of the mixture under investigation. The difficulty in the analysis of this mixture being the presence of MEP which lack UV absorbance so derivatization of this component must be done. TLC is broadly used for the separation and estimation of multicomponent mixtures^39,40^. In this manuscript a TLC chromatographic method was proposed and validated for concurrent analysis of the five components under study.

### Method development and optimization

Meprobamate has no UV absorbance and therefore after developing the system, the spots of ERG, CAF, PHEN and DIP were visualized under UV lamp. Reviewing the published methods, MEP reacts with 1% ethanolic solution of PDAB to give a yellow color that can be seen with the naked eye^37,38^, hence the plate was then sprayed with the mentioned reagent to detect the spots of MEP.

Several experimental trials were done to study different factors that affect the proposed method and to reach optimum separation.

Different eluents were tested with varying solvent composition and fractions, starting with chloroform - methanol, followed by chloroform-acetone then ethyl acetate-acetone; all the previous systems gave bad resolution for the five components under study. Ethyl acetate- methanol was then tried as a developing system in a ratio of (8:2, v/v) and it gave good resolution, however PHEN was at the solvent front due to its partially high lipophilicity. In a trial to reduce the R_f_ of PHEN, a lower polarity solvent mixture of ethyl acetate-hexane was used in ratios of (6:4, v/v) and (7:3, v/v), good resolution was observed. Since, DIP has the least Log P value, it is the most polar one among the studied components and therefore it is more attached to the polar TLC stationary phase and appeared near the baseline. Finally, combination was made for the last two systems consisting of ethyl acetate- methanol- n-hexane in different ratios, the best resolution with reasonable R_f_ for all the five components was obtained when using the ratio of (8:2:3, by volume).

**Fig. 1:**
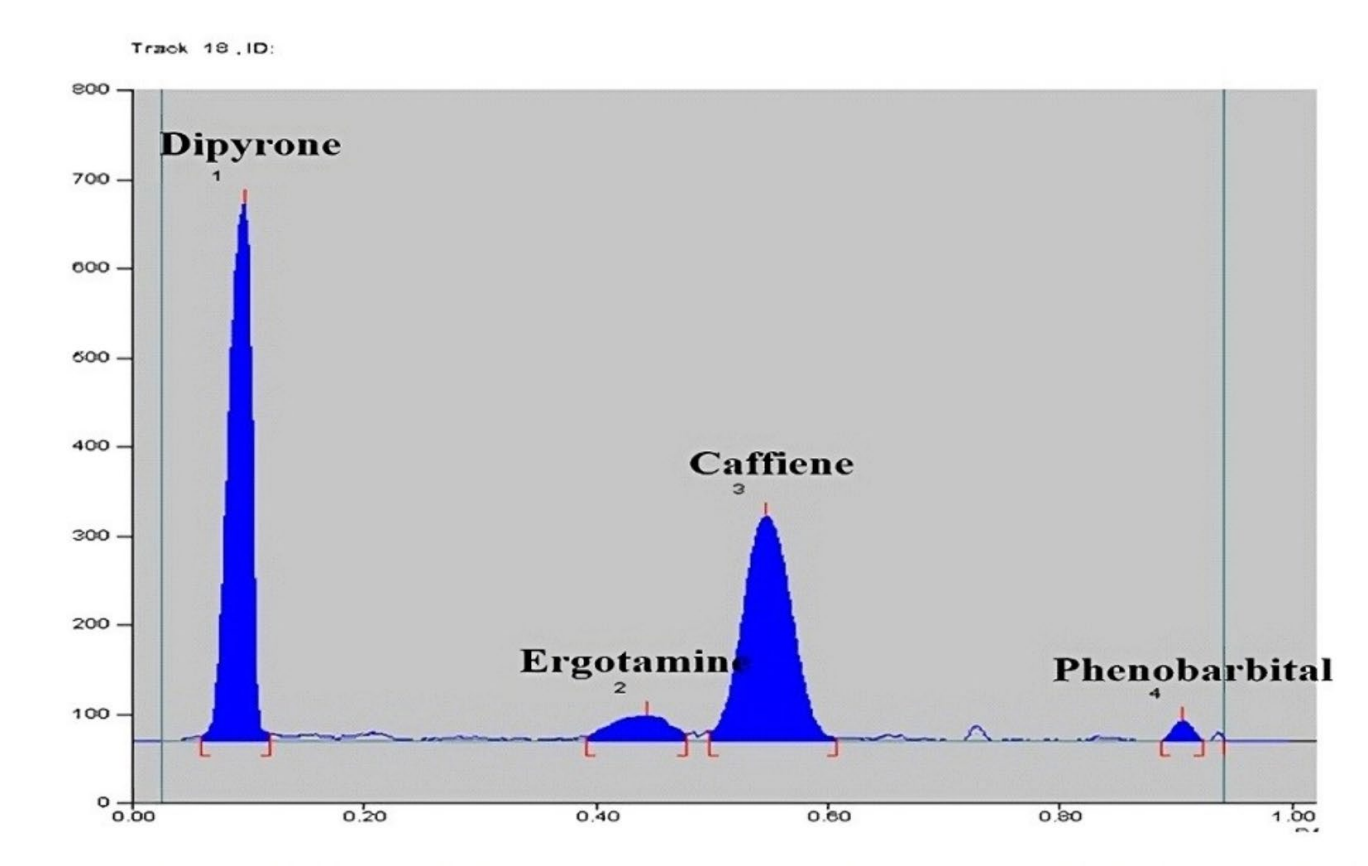
2D densiometric chromatogram of pure drugs scanned at 254 nm of 0.03 µg/band ergotamine, 3 µg/band of phenobarbital, 1.5 µg/band caffeine, 6 µg/band dipyrone.

The highest sensitivity was determined by experimenting with various wavelengths for the four analytes ERG, PHEN, CAF and DIP including 210, 254 and 275 nm. ERG and PHEN were less sensitive at 275 nm and DIP was less sensitive at 210 nm. So the optimum sensitivity was attained at 254 nm with least noise as presented in Fig. 1.

**Fig. 2:**
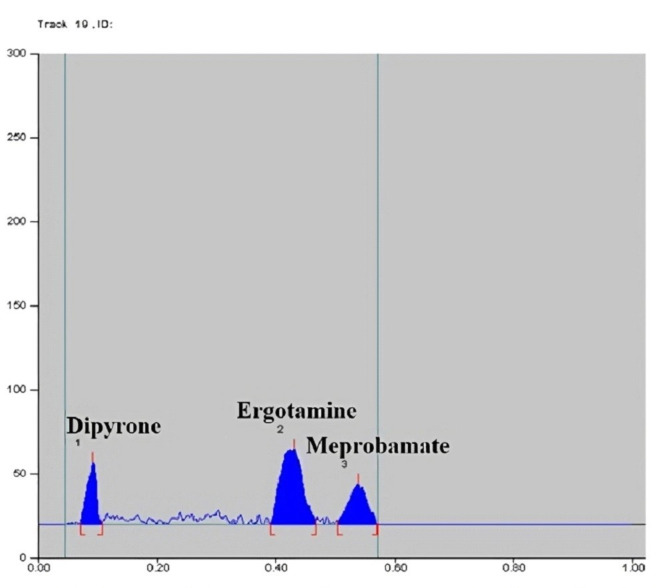
2D densiometric chromatogram of pure drugs scanned at 560 nm of 8 µg/band Dipyrone, 0.04 µg/band ergotamine, 6 µg/band Meprobamate.

Reaction conditions of PDAB with MEP were then optimized; different PDAB concentrations (1%, 0.5% and 0.3%), various temperatures (50, 70 and 110 °C) and several time intervals (15, 20, 30 and 35 min) were tried, the optimum result was obtained by spraying the plates with 0.3% ethanolic solution of PDAB and then drying at 110 °C for 30 min. Scanning the sprayed plates was tried at several wavelengths including 400, 410, 420 and 560 nm for MEP. The best sensitivity and minimum noise were obtained at 560 nm, Fig. 2. Components were well separated at R_f_ of 0.06, 0.36, 0.50, 0.55 and 0.86 for DIP, ERG, CAF, MEP and PHEN, in order.

### Method validation

The developed TLC densitometric approach was validated in agreement with ICH recommendations^35^. Different samples in different concentration ranges were prepared and quantified by the recommended approach. To test linearity; calibration curves representing peak areas against concentrations were plotted. First linear regression equations were applied, where linearity was proved in the intervals of 0.06–2 µg/band regarding ERG and 0.15–5 µg/band regarding PHEN. As for CAF, DIP and MEP, polynomial regression covered wider calibration range with good correlation coefficient in the ranges of 0.3–9 µg/band for CAF, 0.5–10 µg/band for DIP and 1–100 µg/band for MEP. Good correlations were obtained as conferred in Table 1.

**Table 1:** Regression and analytical parameters of thin layer chromatographic method for the determination of the studied components.

Parameter	ERG	PHEN	CAF*	DIP*	MEP*
Calibration range (µg/band)	0.06-1	0.15-5	0.3-9	0.5–10	1-100
Slope	8268.20	664.22	-339.8^a^	125.91^a^	-0.2944^a^
			6108.6^b^	2805^b^	98.667^b^
Intercept	665.78	158.78	1012.30	1267.30	234.01
Correlation coefficient (r)	0.9999	1	0.9999	0.9997	0.9997
Accuracy (mean ± RSD %)	100.20 ± 0.876	100.53 ± 0.709	100.06 ± 0.920	100.07 ± 1.414	98.02 ± 2.008
Precision (RSD%)**					
*Repeatability	1.525	1.782	0.912	1.1039	1.276
*Intermediate precision	1.940	1.886	1.215	1.783	1.324
Robustness (%RSD)***					
Ethyl acetate (8 ± 0.1 mL)	1.447	1.136	0.970	1.525	1.840
Methanol (2 ± 0.05 mL)	1.048	1.567	0.920	0.570	1.620
N-hexane (3 ± 0.05 mL)	0.870	1.180	1.435	1.210	2.008
Wavelength (245 ± 1 nm)	1.135	0.709	1.285	1.414	-

*The linearity was achieved using the polynomial regression equation: A = aX^2^ + bX + C,where a: coefficient 1. b: coefficient 2.

**Precision (RSD of inter- and intraday of three concentrations: ERG (0.03,0.1,1 μg/band),PHEN (0.35,2.5,5μg/band), CAF (0.3,1,7μg/band), MET (0.5,3,10, 7μg/band),MEP(1,30,70μg/band).

***Average of three determination, the %RSD was calculated for the R_f_ values

Process accuracy was carried out by analyzing seven different concentrations for each of the tested drugs, the already calculated regressions were applied for recording the concentrations from which mean percentage recoveries were obtained, as shown in Table 1 all values were close to the true value confirming accuracy of the proposed method. Besides, accuracy was assessed by performing standard addition technique at three scales; 80%, 100%, 120% on dosage form, results proved accuracy of the method and absence of intrusion from additives, Table 2.

**Table 2:** Application of the proposed TLC densitometric method for the determination of Migranil® tablets.

		Taken µg/band	Found ± SD*	Pure added µg/band	Found %**
MARGINAL Tablets Batch No. t095003 Containing1 mg ergotamine tartarate, 10 mg phenobarbital sodium, 50 mg caffeine, 150 mg meprobamate, 200 mg dipyrone	ERG	0.10	102.04 ± 1.55	0.08	101.50
				0.10	103.80
				0.12	100.83
				Mean ± SD	102.04 ± 1.56
	PHEN	1.00	100.73 ± 1.80	0.80	100.16
				1.00	99.30
				1.20	102.75
				Mean ± SD	100.74 ± 1.80
	CAF	1.00	100.65 ± 0.92	0.80	101.12
				1.00	99.60
				1.20	101.25
				Mean ± SD	100.66 ± 0.917
	DIP	4.00	99.91 ± 1.781	3.20	99.46
				4.00	101.87
				4.80	98.39
				Mean ± SD	99.91 ± 1.78
	MEP	15.00	101.77 ± 1.299	12.00	103.17
				15.00	100.60
				18.00	101.55
				Mean ± SD	101.77 ± 1.30

*Average of 6 determinations.

**Average of 3 determinations.

Precision was evaluated by applying the prescribed chromatographic conditions for analysis of three various concentrations for each component on the one day (for testing repeatability) and on three consecutive days (for testing intermediate precision). All the obtained % RSD for all tested drugs by the proposed TLC method were < 2 which proves the precision of the suggested procedure, Table 1.

**Fig. 3:**
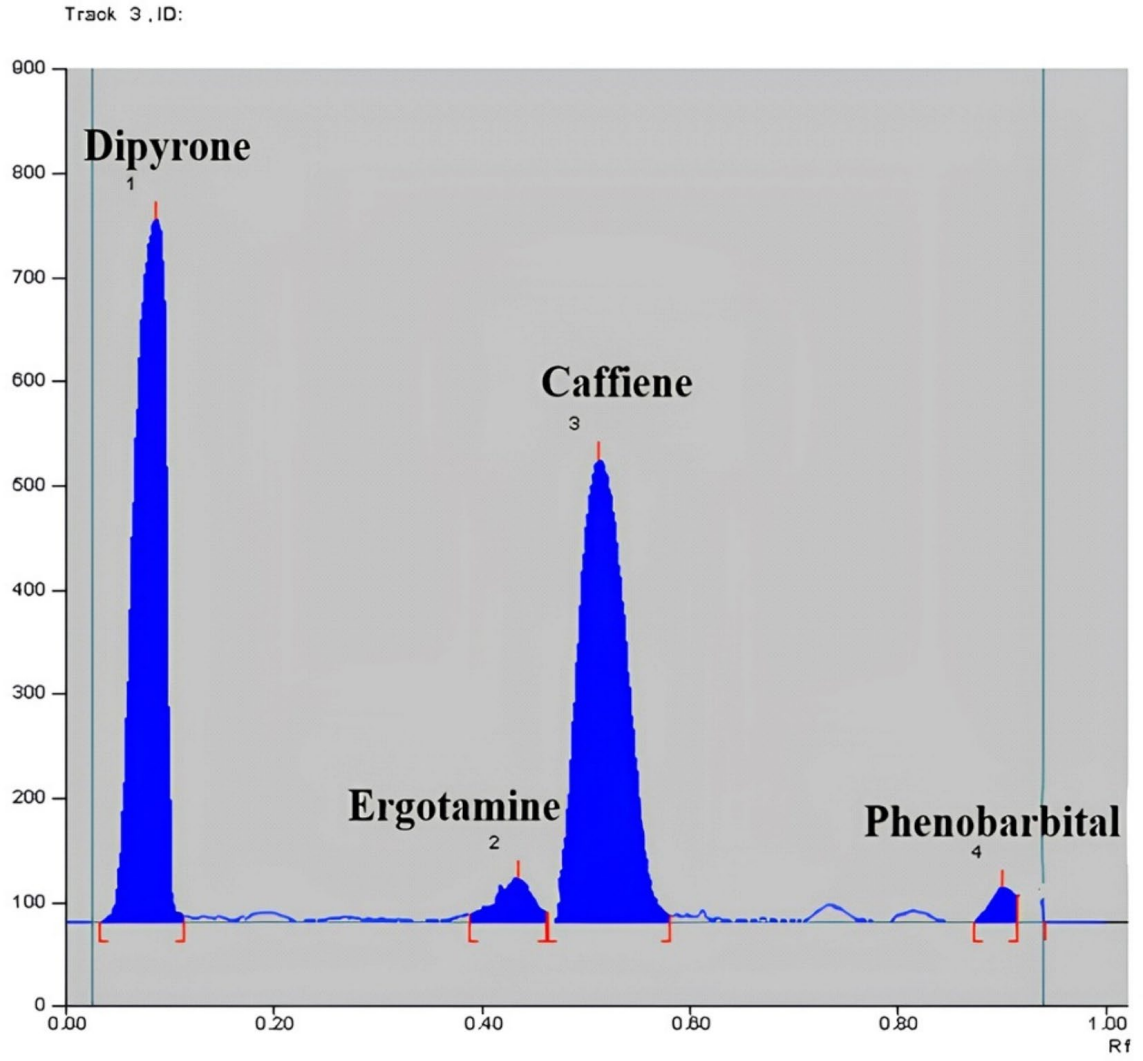
2D densiometric chromatogram of dosage form scanned at 254 nm of 0.1 µg/band ergotamine, 1 µg/band of phenobarbital, 5 µg/band caffeine, 20 µg/band dipyrone.

**Fig. 4:**
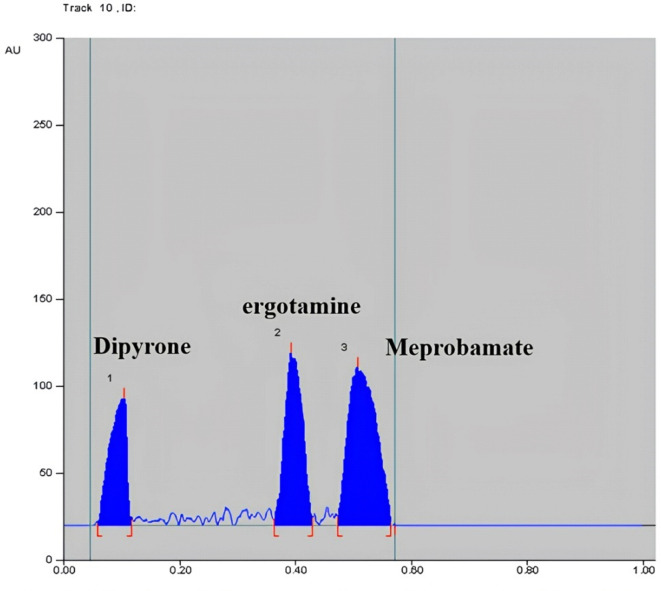
2D densiometric chromatogram of dosage form scanned at 560 nm of 100 µg/band Dipyrone, 0.5 µg/band ergotamine, 75 µg/band Meprobamate.

Method specificity was proved from the densitograms in Figs. 1 and 2, which show complete separation between the studied components. Moreover, specificity was also proved by method application for the intended drugs analysis in their combined tablets, Figs. 3 and 4 where there was no interference from tablets excipients. Moreover, results were within the acceptable values from 90 to 110% which proves absence of intrusion from additives, Table 2. Furtherly, the obtained resolution factors were more than 1.5, ensuring complete separation among the studied components.

Robustness presented as %RSD was tested by minor alterations in eluent composition as well as scanning wavelength and then calculating the R_f_ values after each change. The smaller the %RSD the more robust is the method. Amount of ethyl acetate (8 ± 0.1mL), methanol (3 ± 0.05 mL), and n-hexane (2 ± 0.05 mL) were the intended changes in the mobile phase composition while for scanning wavelength, it was changed by ± 1 nm. The obtained %RSD ranged from 0.57 to 2.008%. These small values ensured that R_f_ values of the studied components were not affected by these small intended changes previously mentioned, confirming robustness of the suggested approach, Table 1.

### System suitability

Different aspects were checked to confirm the consistency of the system. Results in Table 3 confirm peak symmetry as well as good separation among the studied drugs.

**Table 3:** Parameters of system suitability testing of the developed thin layer densitometric method.

Parameters	DIP	ERG			CAF			PHEN	MEP	Reference^46^
Retardation factor (R_f_)	0.06	0.36			0.50			0.86	0.55	–
Resolution (Rs)	5.00		2.20				5.50		–	*R* > 1.5
Retention factor (k)	15.67	1.78			1.00			0.16	0.82	–
Selectivity (α)	8.80			1.78		6.25			–	α > 1
Symmetry factor	1.03	1.00			1.02			1.00	1.05	< 1.5

The presented method was compared statistically with published methods^17,24,38^ using Student t test and F value at p-value of 95%. There was no noteworthy difference between them as shown in Table 4.

**Table 4:** Statistical comparison of the results obtained by the developed TLC densitometric method and the reported methods for determination of ergotamine, phenobarbital, caffeine, dipyrone and meprobamate.

	Proposed TLC method					Reported method				
	ERG	PHEN	CAF	DIP	MEP	ERG^17b^	PHEN^24c^	CAF^17b^	DIP^24c^	MEP^38d^
Accuracy	10.20	100.53	100.06	100.07	98.90	100.55	99.29	100.07	100.66	100.00
SD	0.876	0.709	0.920	1.414	0.566	0.962	1.514	1.053	0.577	0.995
Variance	0.767	0.503	0.846	1.999	0.320s	0.925	2.291	1.109	0.333	0.990
n	7	7	7	7	7	8	4	7	3	5
Student s t-test	0.766 (2.160)^a^	1.544 (2.262)^a^	0.019 (2.179)^a^	0.936 (2.306)^a^	2.227 (2.228)^a^					
F-test	1.098 (4.210)^a^	4.555 (4.760)^a^	1.311 (4.280)^a^	6.003 (19.330)^a^	3.094 (4.530) ^a^					

a. The values between parenthesis are corresponding to the theoretical values of t and F (*p* = 0.05).

b. Reported RP-HPLC method for ergotamine and caffeine using Zobrax C_18_ (150 mm x 2.1 mm) column and gradient elusion was used with a mobile phase with consisting of water (adjusted to pH 3 using orthophosphoric acid): acetonitrile (80:20, v/v) for the first 3 min, (50:50, v/v) for the next 4.5 min, then (80:20, v/v) for the last 2.5 min. Flow rate was 0.7 mL/ min. and UV detection was at 230 nm.

c. Reported RP-HPLC for dipyrone using C18 column and a mobile phase consisting of methanol-water (38, 32, v/v) set at a flow rate of 0.7 mL/min. UV detection was at 254 nm. Whilst for phenobarbital C18 column was used with mobile phase of water-ammonia-methanol (94.5:0.5:5, by volume) and detection was done at 240 nm.

d. Colorimetric determination of meprobamate at 565 nm after its reaction with p-dimethylaminibenzaldehyde.

### Greenness appraisal of the developed method

The fundamental majority of researchers have their primary goal to make analytical methods less harmful to the environment. Green chemistry and its tools are important to evaluate environmental impact of newly developed analytical method and to eradicate hazardous chemicals because their risks have serious effects on climate changes and global warming that the world is suffering from nowadays^41–43^.

It is not easy to use only one metric for the judgment of the overall process as each tool test certain parameters in the method and also some of them are qualitative while others are quantitative. So it is preferred to use more than one tool for evaluation to give a full picture of the impact of any new developed analytical approach.

#### Analytical eco-scale

It is a semi-quantitative method for assessing greenness, it is one of the simplest and most commonly used tool, it depends on computing penalty points for the quantity of reagents or solvents used, energy used, dangers involved, and overall waste produced throughout the analytical processes. Analytical Eco-Scale scores are totaled and ranked. It is represented by a score; the higher the score (close to 100), the better the method^30,42,44,45^. The developed TLC method has a score of 71 which indicates that the method is an acceptable green method, Table 5.

**Table 5:** Results of assessment of greenness profile of the developed TLC method.

Tool	Parameters			Penalty point
Analytical Eco-scale	Reagent	PP of SOLVENT= subtotal pp * number of pictograms * signal word CONSUMED VOLUME = solvent volume in system/no. of spots	Methanol	6
			n-hexane	6
			Ethyl acetate	4
			PDAB	1
			HCl	4
			Ethanol	4
	Instruments	Energy	0 (≤ 60.1 kWh per sample)	0
		Occupational hazard	Analytical process hermitization	0
		Wastes	< 1 mL (g)	1
			No treatment	3
	Total penalty points		29	
	Analytical Eco-scale total score		71	
Agree				
GAPI				
BAGI				
RGB	White, 78.40%			

#### GAPI

This metric is a semi-quantitative one which evaluate the greenness of the whole analytical process from sample gathering to final determination. It is expressed as a pictogram with five pentagrams having color either of green, yellow or red regarding the environmental effect of the method^31,43–45^. The suggested tool was found to have six green fields, six yellow fields and three red ones. The red fields are due to that the method is offline, using the non-green solvent (n-hexane) and there was no waste treatment, Table 5.

#### AGREE

All twelve criteria of green analytical chemistry (GAC) are addressed by AGREE. Both qualitative and measureable findings are grounded on color and number provided by AGREE. Additionally, the outcome of AGREE is easily understood. The twelve criteria are distinctly converted into a 0–1 scale and the final score of AGREE is derived by computation of all the twelve criteria^32,44,45^. As the value approaches one, the procedure becomes more environmentally benign^31,44,45^. Table 5 shows that the newly created approach received a score of 0.62.

#### BAGI

It is one of the more recent assessment tools that is thought to be an addition to the traditional green tools. This metric measures 10 principles to generate a pictogram and a score that describes the practicality and functionality of developed analytical approach. Concerning the attained total score, it is advisable to be ˃ than 60^33,45^. The developed approach achieved a score of 80 ensuring its practicality, Table 5.

#### RGB additive color model

Any analytical method or procedure can be evaluated globally using the RGB additive color model. Three primary colors are used to symbolize three key aspects of the assessed method: productivity/practical effectiveness (blue), conformance with “green” chemistry principles (green), and analytical performance (red). The additive synthesis of the primary colors yields the method’s final color. Additionally, the approach offers a quantifiable metric known as “method brilliance (MB)”^35,45^. Referring to results in Tables 5 and Table S1, it was observed that the method had white color and MB of 78.40%, indicating that the suggested approach can be considered as a good one and can be chosen for several applications.

The overall results of the used metrics confirm the low impact of the developed approach on health and environment.

## Conclusion

A TLC chromatographic method was proposed for the simultaneous quantification of ERG, PHEN, CAF and DIP and MEP in their pure form and their pharmaceutical tablets. The approach has the advantages of using low amounts of solvents, consuming low energy and saving money and time. The drugs were determined with high sensitivity and selectivity. When testing the performance of the method using ICH guidelines, it showed high validity, moreover, the method showed low environmental impact when tested using different green assessment metrics. Moreover, method applicability and whiteness were tested using BAGI and RGB additive color models where the method proved to be the candidate of choice for all applications. This method can be easily applied in any laboratory.

## Electronic supplementary material

Below is the link to the electronic supplementary material.

Supplementary Material 1

## Data Availability

The datasets used during the current study available from the corresponding author on reasonable request.
