# Supplementary material for: TLC densitometric approach for concurrent determination of quinary mixture for treatment of migraine with appraisal to method greenness and whiteness
Source: Sci Rep. 2024 Dec 16;14:30477. doi: 10.1038/s41598-024-79518-5 (PMC11649935; doi:10.1038/s41598-024-79518-5)
Supplement: Supplementary file 1 — Supplementary Material 1 [file 41598_2024_79518_MOESM1_ESM.docx]

**Supplementary table 1. Details of RGB additive color model**

|  |  |  | 2 | | 3 | | 2 | | | 3 | |
| --- | --- | --- | --- | --- | --- | --- | --- | --- | --- | --- | --- |
| **REDNESS (analytical performance)** | | W=4 | **Accuracy (Recovey%)** | | **precision (inter-day %RSD)** | | **linearity (R)** | | | **Sensitivity** | |
|  |  |  |  |  |  |  |  |  |  |  |  |
|  |  |  |  |  |  |  |  |  |  |  |  |
|  |  |  |  |  |  |  |  |  |  |  |  |
| CS: | 81.1% | LAV=33.3 | 100±5% | | ±5% | | 0.999 | | | acceptable | |
|  |  | LSV=66.6 | 100±3% | | ±3% | | 0.9995 | | | satisfactry | |
|  |  | Result | 98.02 | | 1.94 | | 0.9997 | | | satisfactory | |
|  |  | Score (0-100) | 85 | | 85 | | 80 | | | 75 | |
|  |  |  | 2 | | 2 | | 2 | | 2 | | 2 |
| **GREENNESS (safety and eco-friendliness)** | | W=5 | **Organic Solvents/ run** | | **Health/ environmental hazard (pictograms)** | | **Safty (NFPA, inflamability)** | | **Residue (g, mL/run)** | | **Energy consumption** |
|  |  |  |  |  |  |  |  |  |  |  |  |
|  |  |  |  |  |  |  |  |  |  |  |  |
|  |  |  |  |  |  |  |  |  |  |  |  |
|  |  |  |  |  |  |  |  |  |  |  |  |
| CS: | 70.5% | LAV=33.3 | 50 | | 20 | | 4 | | 50 | | 1.5 |
|  |  | LSV=66.6 | 30 | | 10 | | 2 to 3 | | 30 | | ≤1.5 |
|  |  | Result | 20 | | 12 | | 3 | | 25 | | <1.5 |
|  |  | Score (0-100) | 75 | | 60 | | 66.6 | | 72.5 | | 80 |
|  |  |  | 4 | | | | 3 | | | 3 | |
| **BLUENESS (productivity / practical effectiveness)** | | W=5 | **Cost of analysis** | | | | **Sample/hour** | | | **Sample consumption** | |
|  |  |  |  |  |  |  |  |  |  |  |  |
|  |  |  |  |  |  |  |  |  |  |  |  |
|  |  |  |  |  |  |  |  |  |  |  |  |
|  |  |  |  |  |  |  |  |  |  |  |  |
| CS: | 84.9% | LAV=33.3 | ≥20000 | | | | 6 | | | high | |
|  |  | LSV=66.6 | 15000 | | | | 12 | | | moderate | |
|  |  | Result | 5000 | | | | 16 | | | low | |
|  |  | Score (0-100) | 90 | | | | 85 | | | 80 | |
| **FINAL COLOR:** | | | **REDNESS** | | **GREENNESS** | | **BLUENESS** | | **BRILLIANCE (MB):** | | **78.4%** |
| **WHITE** | | | **≥33.3%** | **≥66.6%** | **≥33.3%** | **≥66.6%** | **≥33.3%** | **≥66.6%** |  |  |  |
|  |  |  | yes | yes | yes | yes | yes | yes |  |  |  |
| **#REF!** | | | **Long annotation: 78.4white(81.1/4red-70.5/5green-84.9/5blue)** | | | | | | | | |
|  |  |  |  |  |  |  |  |  |  |  |  |
